# Beyond exercise and appetite: The expanding biology and therapeutic potential of N-lactoyl-phenylalanine

**DOI:** 10.1016/j.jpet.2025.103798

**Published:** 2025-12-18

**Authors:** Olaiya Peter Oni, Barry Scott, Lily C. Schwartz, Tyson J. MacCormack, Mohammed Hankir, Jillian L. Rourke

**Affiliations:** 1 Department of Chemistry and Biochemistry, Mount Allison University, Sackville, New Brunswick, Canada; 2 College of Pharmacy and Nutrition, University of Saskatchewan, Saskatoon, Saskatchewan, Canada; 3 School of Biochemistry and Immunology, Trinity Biomedical Sciences Institute, Trinity College Dublin, Dublin, Ireland

**Keywords:** N-lactoyl-phenylalanine, N-lactoyl-amino acids, Exerkine, Appetite, Carnosine dipeptidase 2

## Abstract

N-lactoyl-phenylalanine (Lac-Phe) has emerged as a signaling metabolite connecting cellular metabolism to systemic physiology. Synthesized through carnosine dipeptidase 2-mediated conjugation of lactate and phenylalanine, Lac-Phe increases acutely in response to exercise and feeding, the primary drivers of its elevation under physiologic conditions. In preclinical models, Lac-Phe acts as a potent regulator of energy balance. Its administration suppresses appetite and reduces body weight in obesity, whereas pharmacologic interventions such as metformin elevate circulating Lac-Phe to produce similar anorexigenic effects. Converging evidence implicates central mechanisms, including inhibition of orexigenic agouti-related peptide neurons, positioning Lac-Phe as a mediator linking peripheral metabolic signals to appetite control. The first human Lac-Phe clinical trial in individuals with obesity began dosing in 2025, evaluating appetite suppression and glucose-lowering effects. Beyond metabolism, Lac-Phe promotes anti-inflammatory macrophage polarization, conferring protection in murine models of colitis and spinal cord injury. Circulating Lac-Phe also rises in conditions such as mitochondrial dysfunction, sepsis, and phenylketonuria, suggesting broader associations with perturbed energy metabolism and systemic stress responses. This review integrates current knowledge spanning molecular mechanisms, physiological regulation, and clinical translation. We examine Lac-Phe biosynthesis, tissue distribution, and regulatory patterns across physiological and disease states, and highlight emerging mechanisms of action in metabolic and inflammatory signaling. Finally, we discuss key knowledge gaps, highlighting the need to define targets, transporters, and tissue sources to shape the next phase of discovery. Collectively, these advances position Lac-Phe at the forefront of exerkine biology and as a promising molecular link between metabolism, immunity, and therapeutic innovation.

**Significance Statement:**

Evidence across molecular, physiological, and translational domains positions Lac-Phe as a promising therapeutic target. This review frames our understanding of Lac-Phe biology—from its biosynthesis to its roles in energy balance and outlines the key questions that will define ongoing discovery.

## Graphical Abstract

**Figure:**
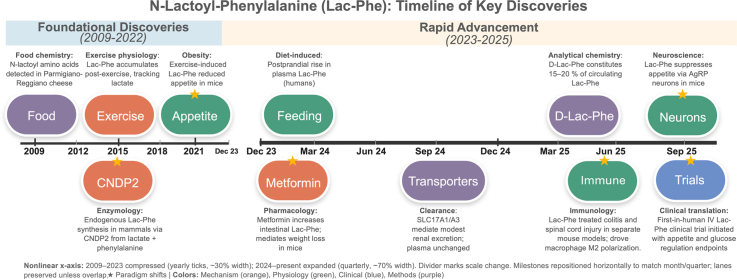

## 1 Introduction

Distinct physiologic and pharmacologic stimuli, including exercise, nutrient intake, and metformin treatment, converge on a common metabolic signal: N-lactoyl-phenylalanine (Lac-Phe), a newly recognized mediator that links cellular metabolism to appetite and systemic energy regulation. This review synthesizes current evidence on Lac-Phe’s biosynthesis and regulation, physiology and pharmacology, candidate mechanisms of action, and translational potential.

Lac-Phe is a member of the N-lactoyl-amino acids, first discovered in fermented foods.^1^^,^^2^ In mammals, untargeted metabolomics revealed that N-lactoyl-amino acids are pseudodipeptides formed by reverse proteolysis of lactate and amino acids.^3^ Specifically, carnosine dipeptidase 2 (CNDP2; also known as CPGL) catalyzes Lac-Phe synthesis from lactate and phenylalanine.^3^^,^^4^

Accelerating discoveries across preclinical and human studies are transforming understanding of Lac-Phe physiology and propelling efforts to map its cellular sources, molecular targets, and signaling architecture (Box 1).^3^, ^4^, ^5^, ^6^, ^7^, ^8^, ^9^, ^10^, ^11^ There remain many important questions in the quest to understand the mechanisms of Lac-Phe physiology, its roles in health and disease, and clinical translation opportunities (Box 1).

Box 1 **Future research questions.**

To fully define the role of Lac-Phe in human physiology and disease—and to harness its bioactivity for treating or preventing metabolic and inflammatory disorders—several key questions remain unanswered.

• Where is Lac-Phe produced in humans and when?
• What are the molecular targets, downstream pathways, and signaling networks engaged by Lac-Phe?
• How do exercise, diet, and pharmacologic interventions (eg, metformin) regulate Lac-Phe synthesis, flux, and tissue distribution?
• What determines individual variability in Lac-Phe responses and sensitivity?
• Can circulating Lac-Phe serve as a biomarker to guide or titrate metabolic interventions such as exercise or metformin therapy?
• Can modulation of the Lac-Phe pathway be harnessed therapeutically to ameliorate metabolic or inflammatory disease?

Here, we synthesize emerging knowledge across molecular, cellular, and systemic levels—from CNDP2 chemistry and kinetics to metabolic regulation, signaling, and neural function—to identify key advances and chart translational paths forward.

## 2 Timeline of discovery and key milestones

**Fig. 1:**
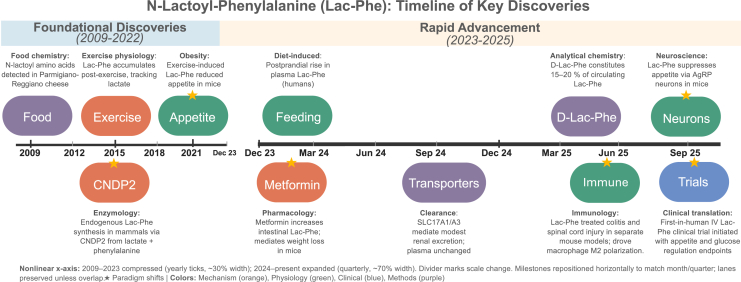
Timeline of Lac-Phe discovery and milestones over the past 2 decades. In 2009–2014, N-lactoyl-amino acids were first detected as a microbial bioproduct of lactic acid bacteria in aged cheese^2^ and shown to be produced enzymatically by a carboxypeptidase.^1^ Jansen et al^3^ discovered that endogenous Lac-Phe production in human cells is mediated through CNDP2 reverse proteolysis. Jansen et al noted Lac-Phe elevations following strenuous exercise, followed by increased levels in mitochondrial disease noted in 2021.^11^ Following this in 2022, Li et al^4^ confirmed that sprint exercise induces Lac-Phe production and that this elevated Lac-Phe suppresses feeding in obese mice. Human postintense aerobic exercise levels were also associated with fat loss in the same year.^12^ In 2024, elevated circulating Lac-Phe levels in humans were reported with feeding and metformin use,^13^^,^^14^ and sepsis.^10^ Renal clearance via solute carrier family 17 member 1/3 (SLC17A1/3) transporters was also established in mice and humans in the same year.^9^ In 2025, advances in detection methods revealed L- and D-Lac-Phe diastereomers^15^ and Lac-Phe signaling was established in immunity and tissue repair,^8^^,^^16^^,^^17^ along with regenerative roles in osteochondral models,^18^ and identification of central mechanisms of neurotransmission.^19^ That year also marked the initiation of the first human trial (NCT06743009), a double-blinded, randomized crossover clinical trial examining the effect of intravenous Lac-Phe on appetite, energy, and glucose homeostasis.^20^ Together, these milestones trace Lac-Phe’s trajectory from food metabolite to bona fide candidate therapeutic axis in metabolism and disease.

Over the past 15 years, the trajectory of Lac-Phe research has evolved from food chemistry to a rapidly expanding field in metabolism, physiology, and pharmacology. The key milestones spanning its discovery, production, exercise regulation, disease links, and emerging translational insights are summarized in Fig. 1.

## 3 Chemistry, biosynthesis, clearance, and measurement

### 3.1 CNDP2-mediated, substrate-driven synthesis

**Fig. 2:**
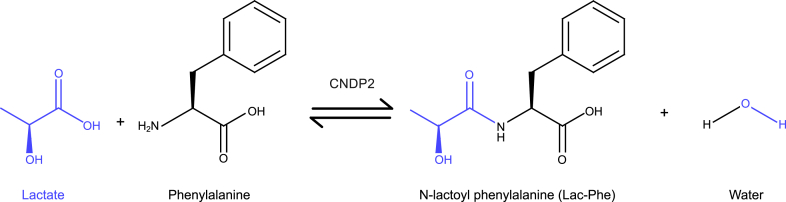
Reverse proteolysis by CNDP2 generates Lac-Phe from lactate and phenylalanine. Schematic representation of the enzymatic synthesis of Lac-Phe by CNDP2. CNDP2 catalyzes the condensation of lactate (blue) and phenylalanine (black) via reverse proteolysis to form the dipeptide Lac-Phe, illustrating the enzymatic coupling of glycolytic and amino acid substrates. Molecular structures were generated using MolDraw.

N-lactoyl-amino acids are made endogenously through the activity of CNDP2, a cytosolic dipeptidase that catalyzes both their formation and degradation (Fig. 2).^3^ Although CNDP2 knockout mice retain detectable circulating Lac-Phe, indicating the presence of alternative biosynthetic routes, CNDP2-mediated catalysis remains the principal pathway for Lac-Phe production.^4^ CNDP2 is highly expressed in several tissues and cell types, with the highest levels in leukocytes (monocytes and macrophages), and epithelial cells of the kidneys and intestines, where it contributes to amide bond catalysis.^21^, ^22^, ^23^ Although predominantly cytosolic, extracellular CNDP2 may arise through exosome export, raising the possibility of extracellular Lac-Phe generation or breakdown.^24^^,^^25^ CNDP2 acts promiscuously to both synthesize and degrade diverse dipeptide and dipeptide-like metabolites, including appetite-regulatory compounds such as *β*-hydroxybutyrate-amino acid conjugates.^26^^,^^27^

Lac-Phe production is generally substrate-limited; however, in some contexts, such as colitis, CNDP2 may become a rate-limiting factor.^3^^,^^13^^,^^16^ Elevated phenylalanine drives Lac-Phe production in phenylketonuria (PKU), whereas lactate predominates in most other contexts.^3^^,^^13^^,^^16^ Supplemental lactate increases Lac-Phe production in macrophage, bladder, and kidney cell lines.^4^ In some organs (eg, skeletal muscle), lactate dehydrogenase, the enzyme responsible for oxidizing glycolytically produced pyruvate to lactate, operates near equilibrium, so enhancing glycolytic flux will increase lactate production.^28^ Any physiologic stimuli that appreciably enhance flux through glycolysis may thus increase Lac-Phe production through the law of mass action, especially when oxygen limits electron transport. Global or regional mismatches between oxygen supply and demand imposed by natural (eg, high altitude and intensive exercise) or pathologic stimuli (eg, ischemia and sepsis), or the inhibition of oxidative phosphorylation (eg, mitochondrial disease and metformin treatment) lead to the accumulation of lactate. Highly oxidative, carbohydrate-dependent tissues such as astrocytes, the inner medulla of the kidney, and certain tumors also tonically produce lactate under aerobic conditions.^29^, ^30^, ^31^ In this context, enhancing lactate catabolism or altering its rate of efflux from tissues through solute carriers will influence substrate supply and thus Lac-Phe production. A comprehensive overview of lactate metabolism^31^ is beyond the scope of this review, but its dynamic, tissue, cell, and context-dependent nature should be considered with Lac-Phe production. The influence of substrate concentrations will require closer examination if alternative mechanisms of Lac-Phe production are identified.

### 3.2 D-Lac-Phe

Lac-Phe research has primarily focused on the L-diastereomer, which CNDP2 generates from L-lactate and phenylalanine. A recent study suggests D-Lac-Phe constitutes ∼15%–20% of circulating Lac-Phe in humans (*n* = 4). This estimate came from specialized reverse-phase liquid chromatography–mass spectrometry (LC-MS) methods that exploit stereoselective interactions to achieve baseline separation, whereas most published “Lac-Phe” measurements likely reflect the combined L + D signal.^15^

The 2 diastereomers are generated through different biosynthetic routes. L-Lac-Phe can arise via CNDP2-mediated condensation of L-lactate and L-phenylalanine.^3^^,^^4^ CNDP2 is stereoselective and neither forms nor degrades D-Lac-Phe.^15^ Accordingly, residual Lac-Phe in CNDP2 knockout models may represent D-Lac-Phe; however, direct quantification of both stereoisomers is required, as the remaining signal could also arise from a second enzyme. D-Lac-Phe forms in the methylglyoxal detoxification pathway, downstream of S-D-lactoylglutathione,^15^ a pathway implicated in hyperglycemic damage, diabetic complications, and age-related metabolic decline.^32^ Though uncharacterized, D-Lac-Phe formation appears enzymatic: D-lactate fails to form D-Lac-Phe in solution but strongly induces it in HEK293 cells.^15^ Their different biosynthetic origins suggest functional divergence, with L-Lac-Phe reflecting lactate metabolism, whereas D-Lac-Phe could reflect methylglyoxal burden. Supporting this functional separation, when humans received racemic lactate (50:50 L:D mixture, both oral and intravenous), circulating L-Lac-Phe increased, whereas D-Lac-Phe remained unchanged.^33^

Unless specified otherwise, herein “Lac-Phe” refers to the L-diastereomer, as most mechanistic work centers on L-lactate–driven pathways. Because human studies typically use nonstereoselective assays, contributions from D-Lac-Phe cannot be excluded. Broader use of stereoselective analytics will be essential to define circulating ratios and distinct functions.

### 3.3 Transport and clearance

Lac-Phe and other N-lactoyl-amino acids are actively secreted following their intracellular synthesis, with significantly higher levels detected in extracellular media compared with inside cells, supporting the role of active release mechanisms.^4^ Although early studies showed a role for the ATP-binding cassette transporter ATP-binding cassette subfamily C member 5 based on its expression patterns and known substrate preferences,^3^ more recent work included ATP-binding cassette subfamily C member 5 among potential exporters of Lac-Phe, but its knockout did not significantly alter circulating levels,^4^ suggesting it is not a major contributor to systemic regulation.

In contrast, the solute carriers solute carrier family 17 members 1 and 3, which are highly expressed in renal proximal tubules, have been identified as the primary transporters responsible for Lac-Phe excretion into urine.^9^ Overexpression of these transporters in vitro significantly increased Lac-Phe efflux, and their genetic ablation in mice led to a marked reduction in urinary Lac-Phe levels—by approximately 30%—without affecting plasma concentrations.^9^ These findings indicate that solute carrier family 17 members 1 and 3 mediate renal clearance of Lac-Phe but do not regulate its systemic levels.

Despite these advances, the primary efflux mechanism responsible for Lac-Phe secretion into the blood remains unresolved. Additional secretory machinery, possibly from other solute carrier family members, the monocarboxylate transporter or organic anion transporters families, may be involved in tissue-specific handling and systemic pharmacokinetics of Lac-Phe.

### 3.4 Systemic and tissue concentrations

Lac-Phe levels display striking variability across physiologic and pathologic contexts (Table 1). In healthy humans, plasma concentrations average ∼25 nM at rest, rising nearly an order of magnitude (∼200 nM) after sprint exercise^4^ and >300 nM during metformin treatment.^13^^,^^14^ Feeding elicits 37%–220% increases depending on nutrient composition.^14^ Among disease states, PKU shows the most pronounced elevations from baseline due to excess phenylalanine,^3^^,^^34^ whereas mitochondrial encephalomyopathy, lactic acidosis, and stroke-like episodes (MELAS) and sepsis also display marked rises linked to broader metabolic stress and lactate accumulation.^10^^,^^11^ In persons with COVID-19, circulating Lac-Phe levels are roughly 4-fold higher in severe cases than moderate cases.^36^^,^^37^ In contrast, glucagon administration lowers plasma Lac-Phe by 38%–42%,^14^^,^^35^ and primary open-angle glaucoma shows a ∼36% reduction, possibly reflecting reduced precursor availability.^38^ Animal studies mirror these trends, with postexercise peaks of 1.5–2.2 *μ*M in mice and racehorses when run to exhaustion,^4^ and diet-driven changes in brain Lac-Phe.^40^ Despite these insights, the absolute range of tissue-specific concentrations in humans is poorly defined; sex and age-related differences are underexplored; and the kinetics of Lac-Phe appearance and clearance, including transporter contributions, remain incompletely understood. Addressing these unknowns will be key to linking concentration dynamics to physiologic function and therapeutic potential.

**Table 1:** Overview of circulating and tissue Lac-Phe concentrations across physiologic and pathologic conditions

Source/Biofluid	Species/Model	Condition/Stimulus	Lac-Phe Concentration	Direction of Change	Notes	References
Plasma	Human	Rest (baseline)	∼25 nM	Not measured	Fasting state baseline	^4^
Plasma	Human	Postsprint/cycling exercise	∼200 nM	↑↑ (∼8× of baseline at rest)	Peaks ∼30–60 min after acute exercise	^4^^,^^12^
Plasma	Human	Metformin-treated	∼320 nM	↑↑ (∼3.35× of premetformin baseline)	Higher level than exercise; independent of glucose levels	^13^^,^^14^
Plasma	Human	Postfeeding	37%–220% ↑ over fasting	↑↑	Magnitude varies by food type: liquid glucose (37%), mixed meal (186%), date fruits (220%)	^14^
Plasma	Human	PKU	Elevated (162 nM from control; 27 nM)	↑↑ (∼6× of reported baseline)	Increase; phenylalanine-driven accumulation	^3^^,^^34^
Plasma	Human	Sepsis	Elevated (∼120 nM reported)	↑↑ (∼6× from ambulatory control)	Plasma marker of mitochondrial dysfunction in septic shock	^10^
Plasma	Human	MELAS	Elevated (∼60 nM)	↑↑ (∼4× from controls)	Possible plasma biomarker of NADH-reductive stress in MELAS	^11^
Plasma	Human	Glucagon administration (low and high)	Reduction (only A.U. increase reported; no *μ*M)	↓↓ (∼42% reduction for low dose and ∼38% for high dose)	Glucagon-driven gluconeogenesis with lactate as substrate	^14^^,^^35^
Plasma	Human	COVID-19	Increase level (no absolute conc. reported)	↑↑ (∼4× increased in severe vs moderate group)	One of the top elevated biomarkers that discriminate severity classes	^36^^,^^37^
Plasma	Human	Primary open-angle glaucoma (POAG)	Decrease level (No absolute concentration reported)	↓↓ (∼36% reduction in POAG group vs healthy controls)	Reduced precursor (phenylalanine) availability and metabolic shift in POAG pathology	^38^
Serum	Human	Dietary habits (healthy)	No baseline reported	Western 7.66 nmol/L; flexitarians 6.91 nmol/L; vegetarians 7.9 nmol/L; vegans 7.4 nmol/L	Lac-Phe concentration reported here correlates positively with glucose and insulin	^39^
Plasma	Mouse and racehorse	Postexercise (running to exhaustion)	∼1.5–2.2 *μ*M	↑↑	Increased from baseline by <0.3 *μ*M in both animals	^4^
Plasma	Mouse	i.p. Lac-Phe administration	∼180 *μ*M	↑↑	Pharmacologic dose, not endogenous	^4^
Brain (hypothalamus/cortex)	Mouse	HCD (basal state)	Low nM baseline; ↑ post-HCD	↑	The increase was time-of-day dependent, independent of calorie intake	^40^

A.U., arbitrary unit; HCD, high caloric diet; i.p., intraperitoneal.

### 3.5 Historical misannotation of N-lactoyl-amino acids

Many mammalian Lac-Phe metabolomics datasets derive from the Metabolon platform, whose historical annotations are directly relevant to this review. Prior to 2022, the platform misassigned several N-lactoyl-amino acids, including Lac-Phe, either as unidentified “X-metabolites” (eg, X-15497) or as 1-carboxyethyl amino acid derivatives (Table 2). Although N-lactoyl and 1-carboxyethyl-amino acids are isobaric and share similar MS fragmentation patterns, they can be distinguished by the liquid chromatography step of LC-MS.^9^ Importantly, datasets generated on the Metabolon platform before 2022, regardless of publication date, may still contain these errors, and all 1-carboxyethyl annotations should be interpreted as N-lactoyl-amino acids.^9^^,^^14^

**Table 2:** Correction of metabolite mislabeling

Unknown	Incorrect ID	Correct ID
X-13529	1-carboxyethylvaline	N-lactoyl-valine
X-15497	1-carboxyethylphenylalanine	N-lactoyl-phenylalanine
X-18889	1-carboxyethylleucine	N-lactoyl-leucine
X-22102	1-carboxyethylisoleucine	N-lactoyl-isoleucine
X-19561	1-carboxyethyltyrosine	N-lactoyl-tyrosine
X-25607	1-carboxyethylhistidine	N-lactoyl-histidine

## 4 Human physiology and pharmacology

**Fig. 3:**
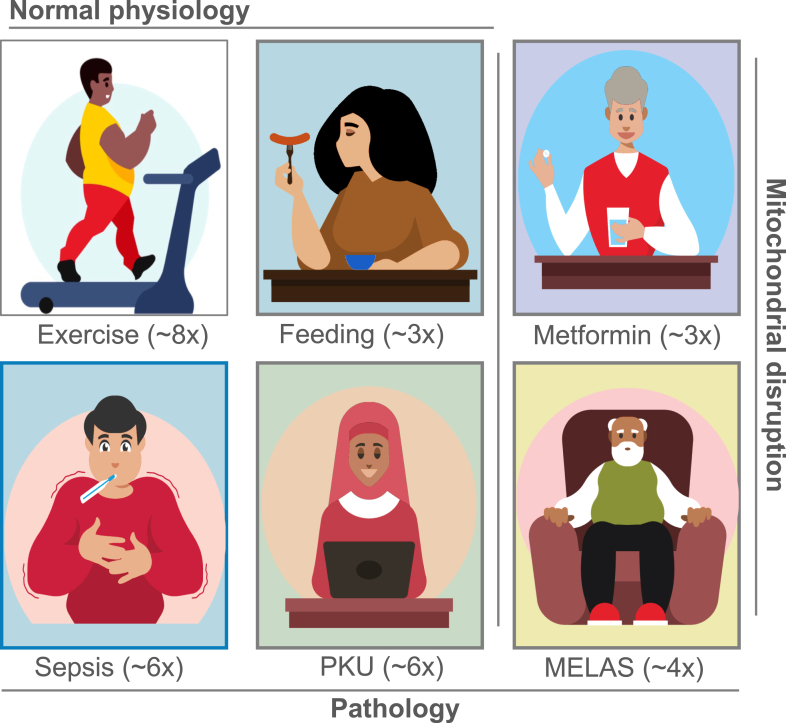
Physiologic, pharmacologic, and pathologic contexts associated with the highest reported blood Lac-Phe increases in humans. Six human contexts shown to produce the most robust elevations in circulating Lac-Phe are illustrated. Approximate fold-changes reflect the upper end of reported human values across studies and are intended to convey relative magnitude rather than precise quantitative comparisons. Normal physiology: high-intensity exercise (∼8×) and postprandial feeding (∼3×). Pathology: sepsis (∼6×), PKU (∼6×), and mitochondrial disease (MELAS, ∼4×). Mitochondrial disruption: metformin (∼3×) and mitochondrial disease (MELAS, 4×) impair mitochondrial respiration, promoting lactate accumulation. Exercise values refer specifically to lactate-producing sprint protocols, and feeding responses to solid or mixed meals rather than glucose alone. See Table 1 and sections *Exercise* through *Other Disease Contexts* for study-specific ranges and references.

The following sections examine Lac-Phe within key physiologic and pharmacologic contexts, including exercise, metformin, feeding, sepsis, metabolic disorders, microbiome interactions, and other disease states (Fig. 3).

### 4.1 Exercise

Lac-Phe has emerged, perhaps intuitively, as one of the most responsive and biologically intriguing exerkines in human metabolism, with high-intensity, lactate-producing exercise identified as its most potent physiological driver. In humans, all-out sprint protocols induce rapid and robust increases in circulating Lac-Phe from ∼25 nM at rest to ∼200 nM within 30–60 minutes postexercise, sustaining elevated levels for several hours.^4^ In contrast, resistance training or moderate endurance exercise elicits only modest rises (≤50 nM), highlighting a likely dose-response relationship between lactate load and Lac-Phe biosynthesis.^3^^,^^4^^,^^12^^,^^41^ Comparable transient surges have been observed in mice and racehorses, where plasma Lac-Phe concentrations reach ∼1.5–2.2 *μ*M following intense running exercise before returning to baseline.^4^ These dynamics strongly suggest lactate, not phenylalanine, as the limiting substrate, consistent with the metabolite’s tight coupling to glycolytic flux.

Exercise-induced Lac-Phe accumulation may play a role in regulating energy balance in humans. Elevated Lac-Phe correlates with exercise intensity (sprint > resistance > endurance) and exercise-induced fat loss.^4^^,^^12^ Higher postexercise (sprint and biking at 80% VO_2_ peak) levels in overweight adults predict greater reductions in visceral and subcutaneous fat during high-intensity training interventions^12^ In diet-induced obese mice, exogenous Lac-Phe administration increases circulating Lac-Phe, suppresses feeding, and reduces body weight and adiposity, with concomitant improvements in glycemic control.^4^ CNDP2 knockout (KO) blunts exercise-induced increases in circulating Lac-Phe, supporting a causal contribution of Lac-Phe to the anorexigenic effect of exercise.^4^

It remains unresolved which tissues predominantly generate Lac-Phe during exercise; skeletal muscle likely supplies most of the lactate, but the Lac-Phe-producing cell types (eg, muscle, epithelial, immune, and adipose) in humans are not defined.^3^^,^^4^ Emerging questions also surround tissue-specific targeting and interindividual variability, including the influence of sex, age, and chronic training status on Lac-Phe kinetics. Beyond appetite, early evidence suggests immunomodulatory links to exercise benefits: exogenous Lac-Phe ameliorated colitis in mice by suppressing nuclear factor-*κ*B signaling and M1 macrophage polarization,^16^ raising the possibility that Lac-Phe may mediate some exercise-induced anti-inflammatory effects and gut barrier effects. Whether Lac-Phe contributes to other adaptations (eg, endothelial function and angiogenesis) remains to be tested in exposure-matched human studies.

### 4.2 Metformin

Metformin is typically the first-line treatment for type 2 diabetes (T2D) because of its ability to lower fasting blood glucose levels with minimal risk of hypoglycemia.^42^^,^^43^ Two parallel studies published in 2024 demonstrated that metformin robustly increases circulating Lac-Phe in humans.^13^^,^^14^ The studies’ 6 cohorts (3 observational and 3 interventional) revealed consistent elevations in Lac-Phe concentrations in both individuals with T2D and those without. In interventional settings, a single-dose study in nondiabetic participants showed acute Lac-Phe increases exceeding 100%.^14^^,^^44^ Longer-term treatment produced sustained rises: in one 12-week trial, Lac-Phe increased by over 80% in both T2D and non-T2D participants,^14^^,^^45^ whereas in another 12-week study of patients with T2D, levels rose by more than 200%.^13^ Intestine specific CNDP2 KO mice demonstrate that metformin-induced Lac-Phe primarily originates from enterocytes in the intestinal epithelium, as opposed to in macrophages.^14^

Beyond its glucose-lowering effects, metformin consistently produces modest weight loss through appetite suppression.^46^^,^^47^ Prior to 2024, several pathways had been proposed to bring about this effect, including growth differentiation factor 15-mediated signaling.^48^^,^^49^ Metformin accumulates 30–300-fold in intestinal tissue,^50^ where inhibition of cellular respiration at mitochondrial complex I shifts metabolism toward glycolysis,^51^^,^^52^ elevating intracellular lactate that drives CNDP2-mediated Lac-Phe synthesis through mass action.^13^

Accordingly, whole body CNDP2 KO mice are resistant to metformin’s appetite-suppressing and weight-reducing effects, although similar findings are needed from intestine specific CNDP2 KO mice to definitively establish gut-derived Lac-Phe as the primary mediator of these actions.^13^ In humans, mechanistic analyses similarly support Lac-Phe as a key mediator of metformin’s appetite suppression and associated weight loss.^13^ Together, these findings suggest that metformin-induced increases in circulating Lac-Phe contribute to the established weight loss and anorexigenic effects of metformin. Given that this effect occurred without alterations in known appetite regulators, such as peptide YY, glucagon-like peptide-1 (GLP-1), or growth differentiation factor 15, it appears that Lac-Phe acts through a distinct anorexigenic pathway.

A key uncertainty is whether Lac-Phe production under metformin treatment is primarily driven in the fasted or fed state. During fasting, metformin increases basolateral intestinal glucose uptake, which could fuel glycolysis and Lac-Phe synthesis.^53^ Conversely, in humans, duodenal metformin alone in the fasted state had little effect on circulating lactate, but prior metformin exposure amplified the lactate surge when glucose was codelivered, implicating luminal substrate, and thus the fed state, as a key driver of metformin-induced lactate (and likely Lac-Phe) generation.^54^ Significantly, the metformin-induced lactate increases correlated strongly with reductions in glycaemia, consistent with metformin-induced increases in glycolytic flux. Given this correlation, Lac-Phe may provide a readout of intestinal glucose clearance via glycolysis during metformin treatment, even though evidence from mice indicates that Lac-Phe does not mediate metformin’s glucose-lowering effects.^13^ Future work will need to establish whether Lac-Phe can serve as a treatment biomarker or mediate metformin’s effects beyond appetite suppression.

In this context, given metformin’s canonical activation of AMP-activated protein kinase (AMPK), it is notable that Lac-Phe increases phosphorylated AMPK in microglia and shows preliminary in silico docking to AMPK*α*1^8^; whether metformin-induced Lac-Phe contributes to AMPK activation in vivo remains unknown. Emerging evidence also links Lac-Phe to immune modulation, but whether this intersects with any anti-inflammatory actions of metformin is unresolved (see Immunomodulation section). Elucidating these pathways could reveal whether Lac-Phe mediates multiple facets of metformin’s pleiotropic effects.

### 4.3 Feeding

Lac-Phe is highly responsive to food intake, much like gut hormones such as GLP-1.^14^^,^^55^ In a recent study,^14^ we reanalyzed untargeted metabolomics data from 3 previously published human feeding studies.^56^, ^57^, ^58^, ^59^ In one cohort,^56^ Lac-Phe levels increased in all 90 paired samples following mixed meals (186% increase), and it emerged as one of the most responsive metabolites to feeding. In a second cohort,^58^ a solid date-fruit challenge produced rises >220%. In a third cohort,^57^ both habitual and high-fat feeding raised Lac-Phe by >160%. Similar postprandial increases have been observed in mice, confirming this response across species.^13^

Postprandial Lac-Phe likely shares the intestinal origin demonstrated during metformin treatment. Oral lactate elicits ∼5-fold larger Lac-Phe increases than intravenous lactate administration at matched plasma lactate levels.^33^ However, intravenous glucose; unlike oral glucose, does not raise the related N-lactoyl-amino acid Lac-Val (Lac-Phe was not assayed).^59^^,^^60^ Although enterocytes are likely the primary source, the microbiome may contribute (see Microbiome). The effect of feeding on intestinal origin Lac-Phe could be probed using intestinal-specific CNDP2 knockout models (eg, by crossing Villin-Cre with floxed CNDP2 mice) as was done with metformin.^13^

Meal composition shapes the magnitude of the Lac-Phe response. As a lactate–phenylalanine conjugate, Lac-Phe could, in principle, report both carbohydrate and protein intake, yet current datasets point to carbohydrate flux as the dominant acute driver; date-fruit (≈70% carbohydrate, ≈1.2% protein) evokes strong responses despite minimal protein.^14^ Carbohydrate type also appears important: in 2 cohorts (an oral glucose tolerance test and a glucose-dominant mixed meal study), Lac-Phe rose only modestly, whereas fructose- or sucrose-containing solid meals produced larger increases.^59^ This pattern aligns with established physiology: fructose generates more postprandial lactate than glucose.^61^^,^^62^ In small-intestinal enterocytes, fructose is preferentially metabolized via ketohexokinase, entering glycolysis downstream of phosphofructokinase and thereby generating substantial local lactate,^63^ and supplying substrate for Lac-Phe synthesis at the site of production. Although these observations require broader validation, they suggest that fructose-containing meals may enhance postprandial Lac-Phe. Identifying which nutrients drive Lac-Phe production could help guide optimization of clinical nutrition for weight gain or loss.

Lac-Phe likely regulates postprandial appetite: it rises reliably after meals, mirrors canonical appetite suppressants such as the gut hormones GLP-1, peptide YY, and cholecystokinin,^54^^,^^64^^,^^65^ and appears enteric in origin. Important distinctions do exist with these gut hormones; however, as their release is regulated by a broad range of stimuli and nutrients that converge to promote enteroendocrine cell depolarization and vesicular exocytosis.^66^ Intestinal-specific CNDP2 knockout experiments, with endpoints of meal size (satiation) and intermeal interval (satiety), would help determine whether, and to what extent, Lac-Phe contributes to satiation and satiety.

### 4.4 Sepsis

In a recent study, circulating N-lactoyl-amino acids, particularly Lac-Phe, were significantly elevated in patients with septic shock.^10^ Lactate is a well established biomarker of impaired cellular respiration and glycolytic dysregulation in severe sepsis.^67^^,^^68^ Lac-Phe may function as a severity biomarker, reflecting excess L-lactate production (with a possible contribution from D-Lac-Phe, as D-lactate is also elevated in sepsis^69^^,^^70^) and reduced renal clearance.^71^^,^^72^ Lac-Phe concentrations provided superior discrimination between survivors and nonsurvivors,^10^ suggesting it could contribute directly to pathophysiology. Two recent studies demonstrate that Lac-Phe can skew macrophage populations toward an anti-inflammatory M2 phenotype.^8^^,^^16^ This raises the possibility that elevated Lac-Phe may exacerbate the immunoparalysis implicated in late sepsis mortality.^73^^,^^74^

### 4.5 Metabolic disease

Targeting the Lac-Phe pathway is of great therapeutic interest as an appetite suppressant and as an anti-inflammatory mediator.^4^^,^^8^^,^^16^ Paradoxically, however, elevated baseline serum Lac-Phe levels are associated with metabolic dysfunction and ill health.^14^^,^^75^ Observational human studies have linked increased serum Lac-Phe concentrations with T2D, insulin resistance, cardiovascular disease, high triglyceride levels, reduced kidney function, and increased risk of sleep disorders, including sleep apnea.^14^^,^^75^, ^76^, ^77^, ^78^, ^79^ Although fold-changes are typically not reported, elevations are likely modest relative to acute physiologic stimuli such as exercise or feeding (Fig. 3).

A recent observational study, which adjusted for metformin treatment, a major confounder often overlooked, found that patients with T2D displayed higher Lac-Phe levels than nondiabetic controls.^75^ Importantly, the elevations were confined to individuals with T2D who also had additional comorbidities, suggesting that higher Lac-Phe may not be a direct feature of diabetes itself but rather a marker of disease burden. Adding further complexity, D-Lac-Phe, also contributes to circulating Lac-Phe.^15^ As the methylglyoxal pathway is upregulated in metabolic disease,^32^ diastereomer-specific analyses are needed to clarify the relative relevance of D- versus L-Lac-Phe in metabolic disease. This paradox—whereby acute Lac-Phe elevation shows therapeutic promise, yet chronic elevation associates with disease—suggests that baseline elevations may reflect a marker of disease burden rather than a causal driver of pathology. Carefully controlled studies are needed to determine the role of elevated Lac-Phe in healthy versus pathologic states and whether elevations arise from impaired clearance or specific disease mechanisms.

Unlike tightly regulated metabolites such as glucose or lactate, Lac-Phe appears to exhibit set points that vary considerably between individuals.^14^ Circulating Lac-Phe correlates with reduced kidney function, suggesting that renal clearance plays an important role in its homeostasis.^75^^,^^76^ The kidney expresses high levels of CNDP2, an enzyme capable of both synthesizing and hydrolyzing Lac-Phe.^3^^,^^80^ Because hydrolysis of Lac-Phe to lactate and phenylalanine is energetically favorable, this raises the possibility that CNDP2 functions mainly in breakdown rather than synthesis within the kidney. Consistent with this, the kidney has been reported to clear peptide-bond–containing metabolites such as glycyl-proline predominantly by hydrolysis rather than simple excretion.^81^ Thus, although a portion of Lac-Phe is excreted in urine,^9^ enzymatic breakdown within the kidney could be the principal clearance route. Impaired kidney function would therefore be expected to limit Lac-Phe breakdown, raising circulating levels and potentially contributing to its disease associations. This hypothesis could be directly tested in a kidney-specific CNDP2 knockout mouse model.

### 4.6 Microbiome

Current evidence indicates that circulating Lac-Phe is primarily governed by host lactate flux and CNDP2 activity (see section *CNDP2-mediated, substrate-driven synthesis*).^13^^,^^33^ In humans, oral, but not intravenous, lactate significantly increases circulating Lac-Phe, and a perfused rat intestine generates Lac-Phe from luminal lactate alone, supporting a role for intestinal processes in Lac-Phe formation.^33^ Intestinal epithelial CNDP2 knockout in mice abolished ∼80% of metformin-induced Lac-Phe, positioning the host epithelium as the primary source under pharmacologic stimulation.^13^^,^^14^

Microbial Lac-Phe synthesis is established in vitro and in food. Fermentation studies confirm Lac-Phe accumulation in cheese and kimchi, with lactic-acid bacteria such as *Lactobacillus rhamnosus* and *Lactobacillus plantarum* capable of synthesizing Lac-Phe via CNDP2-like peptidases.^1^^,^^2^^,^^82^^,^^83^ A gut-resident strain, *Ligilactobacillus salivarius*, also secretes Lac-Phe in vitro, providing proof of principle that resident commensals can produce this metabolite.^84^ These findings suggest that microbial production may supplement host output, particularly under conditions of high luminal lactate. Future studies in germ-free or antibiotic-treated models, coupled with stable isotope tracing of lactate and phenylalanine, are necessary to clarify how much the microbiome contributes to circulating Lac-Phe in vivo.^33^

### 4.7 Other disease contexts

Several pathologic conditions not previously discussed also feature altered Lac-Phe levels, including:

#### 4.7.1 Inherited metabolic disorders

Lac-Phe is elevated in 2 distinct inherited metabolic disorders: PKU and MELAS. Multiple studies report elevated circulating Lac-Phe in PKU, a logical consequence of the characteristic phenylalanine accumulation that defines this disorder.^3^^,^^34^^,^^85^ Although one recent study failed to detect Lac-Phe in pediatric PKU plasma samples,^86^ this likely reflects technical limitations rather than genuine biological absence; the authors relied exclusively on positive-mode LC-MS, whereas N-lactoyl-amino acids are typically detected using negative-mode methods.^4^^,^^14^^,^^15^ Lac-Phe is also elevated in patients with mitochondrial disorders, with higher levels observed in severe MELAS compared with milder mitochondrial dysfunction.^11^ The impaired mitochondrial respiration characteristic of these conditions drives lactate accumulation, providing abundant substrate for Lac-Phe synthesis. This mechanism mirrors complex I inhibitors: both rotenone and metformin impair mitochondrial respiration and similarly elevate Lac-Phe levels.^13^ Although Lac-Phe concentrations correlate with disease severity in both PKU and mitochondrial disorders,^11^^,^^34^ whether it contributes to disease phenotypes or simply reflects underlying metabolic dysfunction remains to be determined.

#### 4.7.2 Inflammatory bowel disease

In contrast to the elevations observed in metabolic disorders, Lac-Phe levels appear reduced in inflammatory colitis.^16^ A recent study found lower serum Lac-Phe and reduced colonic CNDP2 mRNA expression in colitis patients versus controls.^16^ In this study, exercise-induced increases in circulating Lac-Phe levels were concurrent with improved inflammatory outcomes in a dextran-sodium sulfate-induced colitis mouse model, suggesting diminished local Lac-Phe production in colitis may contribute to the inflammatory pathology (see Immunomodulatory section). However, this finding warrants cautious interpretation: the study relied on ELISA-based quantification, an approach vulnerable to crossreactivity and matrix effects when measuring small molecules.^87^^,^^88^ Independent replication of reduced Lac-Phe in colitis using targeted LC-MS/MS, would significantly strengthen confidence in this association. Additional studies are required to determine what mechanistic role Lac-Phe might play in inflammatory diseases.

#### 4.7.3 Cancer and Lac-Phe

Both tumor cells and immune cells in the tumor microenvironment can adopt glycolytic metabolism, producing excess lactate.^89^^,^^90^ CNDP2 is expressed in macrophages that are abundant in tumors.^4^^,^^91^ This suggests Lac-Phe and related metabolites could be generated locally and act in a paracrine fashion on neighboring cells. Such increases are likely to be primarily confined to the tumor milieu, emphasizing the need for tissue-specific measurements rather than reliance on serum analyses that may underestimate local concentrations.

Clinical evidence for altered Lac-Phe in cancer is limited but suggestive. Serum Lac-Phe has been reported to rise several years before glioma diagnosis.^92^ In clear cell renal cell carcinoma, the most common kidney cancer, CNDP2 expression was the highest among 33 tumor types examined.^93^ Elevated N-lactoyl-leucine was detected in clear cell renal cell carcinoma cell lines compared with nontumor controls (Lac-Phe was not measured), and a small patient cohort (*n* = 5) showed a trend toward higher serum levels of this metabolite.^94^ These findings suggest that N-lactoyl-amino acid concentrations may be elevated in certain cancers.

Mechanistic studies highlight both the promise and the complexity of linking Lac-Phe biology to cancer progression. In clear cell renal cell carcinoma, one group proposed that CNDP2-mediated conjugation of lactate to amino acids acts as a tumor-suppressive mechanism by exporting N-lactoyl-amino acids and thereby depleting intracellular amino acid pools.^93^ Lactate-treated CNDP2-high cells showed reduced amino acid availability in vitro and in xenograft models. However, the N-lactoyl-amino acids themselves were not directly quantified, limiting the strength of this conclusion. By contrast, another report described extracellular CNDP2 supporting tumor growth by hydrolyzing dipeptides, substrates distinct from N-lactoyl-amino acids, and thereby supplying amino acids to cancer cells.^25^ These opposing findings underscore the context-dependent effects of CNDP2,^95^^,^^96^ and highlight the difficulty of attributing its cancer phenotypes specifically to Lac-Phe.

## 5 Mechanisms and therapeutic applications

Although Lac-Phe’s regulation by exercise, feeding, and metformin is increasingly clear, its mechanisms of action and therapeutic applications span diverse physiological systems. The following sections integrate emerging mechanistic insights with therapeutic potential.

### 5.1 Central neural integration—gut sensing, brainstem, arcuate nucleus, and meal-frequency regulation

The brain circuits through which Lac-Phe modulates feeding behavior have only recently begun to be defined. The hindbrain nuclei engaged by Lac-Phe—the area postrema, nucleus tractus solitarius (NTS), and parabrachial nucleus—belong to the canonical gut–brain circuitry that integrates visceral and circulating satiation cues to influence meal size and communicate with hypothalamic regulators of energy balance.^7^^,^^97^, ^98^, ^99^ Using c-Fos immunohistochemistry, Lac-Phe (50 mg/kg i.p.) was shown to activate neurons in the hindbrain area postrema and NTS as well as downstream neurons in the parabrachial nucleus in mice.^27^ Although the neurochemical identity of these neurons was not identified, it is unlikely that Lac-Phe activates aversive tyrosine hydroxylase (TH)-expressing neurons in the NTS or calcitonin-gene-regulated peptide-expressing neurons in the parabrachial nucleus,^100^ because Lac-Phe does not cause aversive responses in mice.^4^ Despite Lac-Phe’s activation of various hindbrain neuronal populations, chemogenetic inhibition of NTS neurons does not prevent its acute appetite-suppressing effects.^19^ This contrasts with GLP-1R agonists, which require hindbrain neurons for their effects on energy balance and aversion.^101^ The fact that Lac-Phe does not engage hindbrain aversive circuitry to suppress appetite lends further support for its potential use to treat obesity.

Beyond the hindbrain, c-Fos immunohistochemistry revealed Lac-Phe activates neurons in various hypothalamic regions, including the arcuate nucleus (ARC), ventromedial hypothalamus, dorsomedial hypothalamus, and particularly the paraventricular nucleus of the hypothalamus (PVH).^25^ The effect of systemically administered Lac-Phe on hypothalamic neurons is likely direct, since bath application of Lac-Phe (1–50 *μ*M) to mouse hypothalamic slices robustly activates anorexigenic ARC proopiomelanocortin neurons and inhibits orexigenic agouti-related peptide (AgRP) neurons via opening ATP sensitive potassium channels as determined by patch-clamp electrophysiology.^19^ Whether Lac-Phe actions on these channels are direct or secondary to engagement of a membrane or intracellular target remains to be tested. Importantly, these concentrations of Lac-Phe are within the range measured in the bloodstream upon intraperitoneal administration in mice,^4^ and circulating Lac-Phe in principle accesses ARC neurons because of its proximity to the fenestrated capillaries of the median eminence. Correspondingly, fiber photometry experiments on mice expressing an intracellular Ca^2+^ sensor (GCaMP6) in ARC AgRP neurons revealed that systemic administration of Lac-Phe robustly inhibits this orexigenic population of neurons in vivo. Moreover, diphtheria toxin-mediated ablation of ARC AgRP neurons and chemogenetic inhibition of PVH neurons occluded the appetite-suppressing effects of Lac-Phe, providing strong evidence that an ARC^Agrp^→PVH circuit is targeted by this circulating metabolite.^19^ The robust Lac-Phe activation of PVH neurons is potentially downstream of increased *α*-melanocyte-stimulating hormone release from ARC proopiomelanocortin neurons binding to postsynaptic melanocortin 4 receptors in the PVH.^102^ Notably, Lac-Phe does not appreciably influence the release of other factors that regulate food intake and body weight, such as leptin, ghrelin, peptide YY, GLP-1, or growth differentiation factor 15,^4^^,^^13^ further suggesting it has direct effects on the brain.

An important consideration to make when assessing the Lac-Phe targeting of brain feeding circuits is the Lac-Phe source. For example, the T2D drug metformin has been shown to reduce food intake via increasing the synthesis of gut-derived Lac-Phe.^13^ A strong possibility is that under these conditions, anorexigenic vagal afferent neurons and/or spinal afferent neurons that innervate the gut are activated in a paracrine manner by the high local concentrations of Lac-Phe. One straightforward way of testing this is to administer metformin orally or Lac-Phe intraperitoneal to mice with or without vagotomy or capsaicin-induced sensory nerve deafferentation. As an exerkine, Lac-Phe is also likely produced in peripheral tissues by CNDP2+ macrophages and epithelial cells following surges in muscle-derived lactate during strenuous exercise.^5^ Exercise-induced and intraperitoneal Lac-Phe would be expected to activate similar brain feeding circuits, although this remains to be formally shown. Finally, there is evidence that feeding increases hypothalamic Lac-Phe levels in rats as determined by using stable isotope dilution LC-MS/MS on whole hypothalamic blocks.^40^ The cellular source of Lac-Phe in the brain is most likely microglia, since these cells are enriched for CNDP2 transcript.^103^ Future studies are needed to determine if and how feeding induces Lac-Phe in hypothalamic microglia and the downstream target neurons.

### 5.2 Lac-Phe and inflammatory diseases

**Fig. 4:**
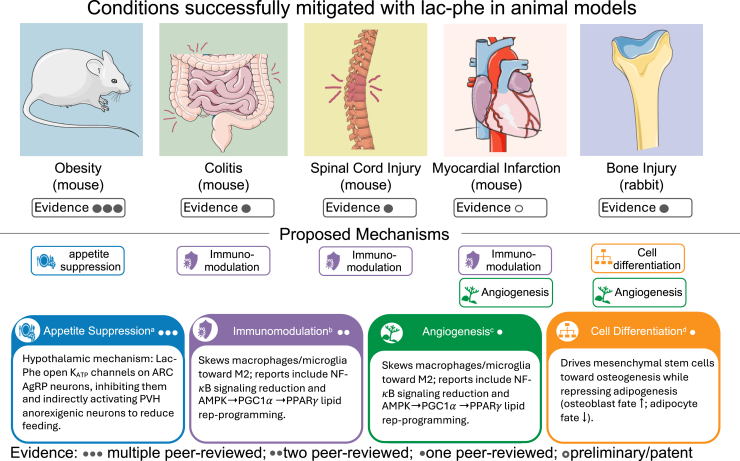
Promising therapeutic benefits of Lac-Phe in animal models of disease and injury. Panels illustrate disease and injury contexts in which exogenous Lac-Phe improved outcomes versus vehicle or appropriate controls in animals (species noted in panel captions). Colored capsules beneath each condition indicate proposed mechanisms (≥1 per condition), matching the mechanism cards: blue, appetite suppression; purple, immunomodulation; green, angiogenesis; orange, cellular differentiation. For example, in spinal cord injury and myocardial infarction, Lac-Phe–mediated immunomodulation is implicated. Administration and dose (by model). ● Obesity (diet-induced): Lac-Phe intraperitoneal injection, 50 mg/kg/d (diet-induced obesity mice; repeated intraperitoneal dosing reduced body weight).^4^ ● Colitis (dextran-sodium sulfate): Lac-Phe intraperitoneal injection, 0.2 mg/kg/d for 21 days.^16^ ● Spinal cord injury (contusion): Lac-Phe tail-vein injection daily for 1 week; main effective dose 20 mg/kg.^8^ ● Myocardial infarction (left anterior descending ligation, patent): Lac-Phe intraperitoneal injection, 20 mg/kg for 1 week.^104^ ● Osteochondral defect (bone/cartilage): Lac-Phe not injected; delivered via a Lac-Phe–loaded silk methacrylate coating on a 3D-printed bioactive-glass scaffold.^18^ All cited preclinical models included appropriate control or vehicle-treated comparators, except the myocardial infarction patent, which compared Lac-Phe–treated mice to untreated myocardial infarction controls. All dosing information is derived from the original studies.^4^^,^^8^^,^^16^^,^^18^^,^^104^ SilMA, silk fibroin methacryloyl. ^a^Effect versus mechanism: Appetite suppression is replicated across multiple studies; the hypothalamic K_ATP_–AgRP→PVH node is from one primary study. ^b^Convergent phenotype, differing nodes: M2 bias replicated in 2 peer-reviewed models (colitis, spinal cord injury); proximal node differs (colitis emphasizes nuclear factor-*κ*B (NF-κB) ↓, spinal cord injury highlights AMPK→peroxisome proliferator-activated receptor gamma coactivator 1-alpha→peroxisome proliferator-activated receptor-*γ* [PPAR*γ*]). These are not mutually exclusive. ^c^Evidence scope: One peer-reviewed osteochondral biomaterial study plus a myocardial infarction patent, both showing in vitro endothelial assays; no in vivo angiogenesis demonstrated to date. ^d^Scope of evidence: One peer-reviewed scaffold study shows osteogenesis ↑/adipogenesis ↓ in mesenchymal stem cells with PPAR*γ* and CCAAT/enhancer-binding protein *α* downregulated. SilMA, silk fibroin methacryloyl. Images adapted from Servier Medical Art (https://smart.servier.com), licensed under CC BY 4.0 (https://creativecommons.org/licenses/by/4.0/).

Emerging evidence suggests that Lac-Phe could function as a potent immunomodulatory agent across multiple disease contexts, with therapeutic effects consistently linked to macrophage reprogramming from proinflammatory to anti-inflammatory phenotypes.^8^^,^^16^^,^^104^ This immunomodulatory activity opens new lines of investigation for Lac-Phe’s translational potential beyond appetite control (Fig. 4).

In colitis, human studies reveal decreased circulating Lac-Phe levels during active disease.^16^ At the same time, exogenous Lac-Phe administration in mouse models—effective at intraperitoneal doses as low as 0.2 mg/kg, roughly 250-fold lower than the 50 mg/kg used in the seminal study for appetite suppression^4^—alleviates disease severity through reduced macrophage infiltration into gut tissue and polarization toward M2-like, proresolving macrophages.^16^ Lac-Phe administration directly suppressed nuclear factor-*κ*B activation and inhibited M1 macrophage polarization, attenuating inflammation.^16^ Although the upstream target and effectors remain unknown, this provides the first indication that Lac-Phe can modulate canonical immune pathways, supporting its role as more than a biomarker and raising the possibility of paracrine or endocrine immunometabolic signaling.

Similarly, in a spinal cord injury model, either exercise or Lac-Phe administration (20 mg/kg) reduced the accumulation of lipid-laden, foam-cell-like macrophages at the injury site while promoting a shift toward M2-like macrophages and microglia, changes linked to enhanced functional recovery.^8^ The Lac-Phe administration aligned with those previously shown to influence appetite and colitis outcomes; however, the atypical Lac-Phe quantification method used to detect exercise-induced Lac-Phe limits interpretation of the obtained endogenous levels. This methodological consideration does not detract from the observed therapeutic benefit of exercise or exogenous Lac-Phe, but does constrain inferences about the contribution of exercise-induced Lac-Phe in this context. A nonpeer-reviewed patent reports that Lac-Phe lowers interleukin 6 and tumor necrosis factor *α* in lipopolysaccharide-stimulated RAW 264.7 macrophage-like cells and proposes macrophage-mediated proresolving activity contributing to improved myocardial infarction outcomes in Lac-Phe–treated mice.^104^ Additionally, bone regeneration studies using Lac-Phe-containing scaffold matrixes report reduced inflammation and enhanced remodeling. However, the primary anti-inflammatory effects in these studies were attributed to codelivered CK2.1 rather than Lac-Phe directly.^18^

A consistent theme is emerging: Lac-Phe appears to skew macrophages from proinflammatory M1-like states toward proresolving M2-like phenotypes across multiple tissue contexts. This immunomodulatory capacity may prove protective in inflammatory diseases such as colitis and spinal cord injury.^8^^,^^16^

Lac-Phe immunomodulation could potentially also be maladaptive in contexts requiring robust immune responses, such as late-stage sepsis^10^ or cancer surveillance. Notably, a Mendelian randomization analysis reported a causal association between higher genetically predicted Lac-Phe and increased rosacea risk, underscoring that Lac-Phe elevation may not be uniformly beneficial across inflammatory diseases.^105^ Future research must clarify the actual signaling mechanisms, establish the conditions under which Lac-Phe is beneficial versus potentially harmful, and develop more rigorous methodological approaches for studying this promising therapeutic target.

### 5.3 Lac-Phe and angiogenesis

Because angiogenesis is a hallmark of exercise adaptation and lactate can stimulate endothelial growth,^106^^,^^107^ it is plausible that Lac-Phe contributes to vascular remodeling. Supporting this hypothesis, a bone-remodeling study reported that Lac-Phe promoted endothelial proliferation, migration, tube formation, and angiogenesis-related gene expression, with the most potent effects when delivered via a bioactive-glass scaffold.^18^ In addition, nonpeer-reviewed patent filings describe proangiogenic activity in human umbilical vein endothelial cells and link these effects to improved recovery after myocardial infarction.^104^ Notably, rosacea is also marked by vascular dysregulation and remodeling (including angiogenesis)—suggesting that Lac-Phe may influence angiogenesis in this context.^105^ These observations suggest a potential angiogenic role for Lac-Phe, but dedicated mechanistic and in vivo vascular studies are needed to establish causality and physiological relevance.

### 5.4 Lac-Phe and adipose tissue

Recent evidence hints at possible direct roles of Lac-Phe in lipid handling and adipose tissue regulation. Similar to the reduction in lipid load in microglia,^8^ Lac-Phe exposure during mesenchymal stem cell differentiation reduces lipid accumulation and downregulates adipogenic transcription factors including peroxisome proliferator-activated receptor-*γ* and CCAAT/enhancer-binding protein *α*, ADIPOQ, and LPL, shifting differentiation toward osteogenesis.^18^ This finding raises the possibility that Lac-Phe can directly reduce adipose mass. CNDP2 is present in white adipose tissue and rises with fat-reducing interventions such as caloric restriction and t10-c12-CLA,^108^ indicating adipose tissue may serve as both a source and target of Lac-Phe. Consistent with this, adipocyte AMPK*α*2 shapes diurnal metabolite rhythms, with Lac-Phe rhythmicity lost in adipocyte-specific AMPK*α*2 knockdown,^17^ implicating adipose tissue in circadian control of systemic Lac-Phe. Lac-Phe may thus serve an autocrine/paracrine role in adipose tissue, modulating fat mass beyond its central anorexigenic effects.^19^ Elucidating the involvement of CNDP2 in this context may help to refine current obesity treatments.

Clinically, intense exercise (bicycling at 80% VO_2_ peak) induced Lac-Phe correlates with reduced subcutaneous and visceral fat mass in the abdomen, the depots most strongly linked to metabolic risk.^12^^,^^109^ Activation of thermogenic brown adipose tissue is a plausible mechanism for Lac-Phe–mediated weight loss, but this appears unlikely as Lac-Phe fails to acutely increase oxygen consumption in metabolic cage experiments in diet-induced obese mice.^4^ It will be important to assess whether chronic Lac-Phe treatment affects whole body energy expenditure and promotes browning of white adipose tissue, a process known to enhance weight loss in preclinical models.^110^ Given the endocrine role of adipose in coordinating appetite, insulin sensitivity, and inflammation,^111^ Lac-Phe’s influence on fat depots may represent a second axis of action, linking local tissue remodeling with systemic metabolic health.

### 5.5 Candidate molecular targets

Despite Lac-Phe’s emerging roles in metabolism and appetite regulation, its precise molecular mediators, modes of action, and downstream cellular targets remain undefined. Identifying the intracellular or membrane receptors and the cellular effector pathways that mediate Lac-Phe signaling remains a central challenge—and a critical next step for advancing the field.^5^

Lac-Phe may exert cell-intrinsic effects if transported or retained within the cytosol. To our knowledge, no intracellular proteins have yet been definitively shown to bind Lac-Phe directly. Network pharmacology identified Lac-Phe as a possible protective metabolite in ovarian cancer, and in silico modeling predicted weak binding (−5.7 kcal mol^−1^) at catenin *β*-1 (*β*-catenin)^112^ with no functional validation. Similarly, binding to AMPK*α*1 was proposed (−5.8 kcal mol^−1^)^8^ but the modeling approach and proposed binding site await independent validation. Evidence is mounting for a Lac-Phe/AMPK signaling axis. AMPK KO modulates Lac-Phe signaling in spinal microglia and AMPK*α*2 knockout in adipocytes reduced serum Lac-Phe rhythmicity.^8^^,^^109^ This is consistent with the known role of AMPK in mediating the peripheral tissue effects of metformin. In contrast, AMPK activation is unlikely to underlie Lac-Phe’s well established effects in hypothalamic neurons, as it would be expected to increase rather than suppress appetite in these neurons.^7^^,^^113^ One might reasonably assume that AMPK involvement, if any, is likely limited to cell metabolism and stress and not appetite regulation in the brain.

Two context-dependent modes of Lac-Phe signaling have been proposed for Lac-Phe as an extracellular signal activating membrane G-protein-coupled receptors (GPCRs): (1) an endocrine route targeting central appetite control with nanomolar to low micromolar Lac-Phe sensitivity, and (2) a paracrine route at production sites where local concentrations may reach millimolar levels.^5^ This dual-mode hypothesis parallels the paracrine action of succinate at its cognate GPCR.^114^^,^^115^ Lac-Phe’s precursors reinforce mechanistic plausibility for this GPCR hypothesis. Extracellular lactate signals through GPCRs such as GPR81 (HCAR-1) and GPR132, influencing metabolic and immune programs.^116^, ^117^, ^118^, ^119^ Phenylalanine also activates the Class C GPCR calcium-sensing receptor to regulate satiety hormone secretion from the gut.^120^ Phenylalanine may also engage distinct Class A GPCRs, with unpublished data suggesting it acts as a promiscuous endogenous activator of arrestin recruitment across multiple Class A GPCRs.^121^

Consistent with a GPCR hypothesis, molecular docking shows that Lac-Phe and other N-lactoyl amino-acids (eg, Lac-Leu, Lac-Tyr, Lac-Trp, and Lac-Ile) can bind taste receptors, including bitter, sweet (T1R2/T1R3), kokumi (CaSR, T1R1/T1R3), and umami receptors, suggesting possible roles in nutrient sensing.^122^^,^^123^ Our group has generated prospective GPCR-centric evidence: a JPET 2024 abstract reports 5 blinded Class A GPCR hits for Lac-Phe identified via PRESTO-Tango *β*-arrestin screening.^6^ These hits show concentration-dependent activation with micromolar EC_50_ values consistent with putative paracrine signaling.^6^ Although these candidates await comprehensive EC_50_ determination, direct-binding studies, pharmacologic profiling, mutational mapping, and in vivo receptor knockout validation,^124^ they offer potential for identifying druggable Lac-Phe targets as one of medicine’s most druggable receptor classes.^125^, ^126^, ^127^

These data expand Lac-Phe’s putative signaling landscape, but stronger mechanistic evidence is needed in all cases to establish these proposed pathways. Target identification remains a critical next step for enhanced mechanistic understanding, structure-activity relationship development, and rational drug design or mimetic analogs. Even if target identity lags, ligand-centric medicinal chemistry, phenotypic assays, and circuit-mapping offer interim translational routes.^124^^,^^128^ Lac-Phe thus stands at a pivotal inflection point-akin to ghrelin, succinate, and short-chain fatty acids—where target discovery will advance the trajectory of future therapeutic applications.

## 6 Conclusions

The field of Lac-Phe research has now reached a critical juncture. In mid-2025, the first-in-human clinical trial began recruiting participants.^20^ This double-blinded, randomized crossover study will assess whether intravenous Lac-Phe can modulate appetite and glucose regulation, with additional endpoints including gastric emptying and triglyceride levels. This milestone marks the transition from preclinical promise to translational testing. Yet despite this progress, many challenges and unanswered questions remain.

Currently, low oral bioavailability limits exogenous Lac-Phe delivery,^4^ because direct oral dosing is likely ineffective owing to peptide bond hydrolysis in the gastrointestinal tract.^4^ A significant hurdle is the development of orally active Lac-Phe mimetics. Other fundamental priorities include identifying the canonical transporter responsible for exporting Lac-Phe from producing cells and those that might allow uptake in target cells. Equally important is defining how Lac-Phe is cleared once generated, particularly the relative contributions of urinary excretion versus enzymatic degradation. Further work must also clarify which enzymes beyond CNDP2 contribute to Lac-Phe synthesis and breakdown, disentangle the distinct roles of its D- versus L-diastereomers, and determine whether Lac-Phe signals through intracellular, extracellular, or multiple receptor systems. Together, these questions frame the central agenda for the next phase of Lac-Phe research.

## Conflict of interest

All authors declare no conflicts of interest.
